# Effects of honey saccharide supplementation on growth performance, amylase enzyme activity, gut microvilli, and microbiome in *Cyprinus carpio*

**DOI:** 10.14202/vetworld.2025.228-237

**Published:** 2025-01-30

**Authors:** Yani Aryati, Ekorini Farastuti, Lili Sholichah, Isti Koesharyani, Lila Gardenia, Early Septiningsih, Muhamad Yamin, Parwa Oryzanti, Dewi Puspaningsih, Desy Sugiani

**Affiliations:** 1 Research Center for Applied Microbiology, National Research and Innovation Agency, Jl. Raya Jakarta-Bogor Km. 46, Cibinong 16911, West Java, Selatan, 15418, Indonesia; 2 Faculty of Agriculture, Djuanda University, Jagorawi Toll Rd No.1, Ciawi, Bogor Regency, West Java 16720, Indonesia; 3 Research Center for Fisheries, National Research and Innovation Agency, Jl. Raya Jakarta-Bogor Km. 46, Cibinong 16911, West Java, Selatan, 15418, Indonesia; 4 Research Center for Veteriner, National Research and Innovation Agency, Jl. Raya Jakarta-Bogor Km. 46, Cibinong 16911, West Java, Indonesia; 5 Research Center for Conservation of Marine and Inland Water Resources, National Research and Innovation Agency, Jl. Raya Jakarta-Bogor Km. 46, Cibinong 16911, West Java, Indonesia; 6 Research Center for Ecology and Ethnobiology, National Research and Innovation Agency, Jl. Raya Jakarta-Bogor Km. 46, Cibinong 16911, West Java, Selatan, 15418, Indonesia

**Keywords:** amylase, gut microbiome, honey saccharides, microvilli, prebiotics

## Abstract

**Background and Aim:**

Prebiotics, such as saccharides in honey, play a crucial role in improving gut microbiota, digestion, and immune function. This study evaluates the effects of Kapok flower honey saccharides on growth performance, digestive enzyme activity, intestinal morphology, and gut microbiota in common carp (*Cyprinus carpio*).

**Materials and Methods:**

A completely randomized design was implemented with four honey supplementation levels (0% control, 0.5%, 0.75%, and 1%) applied to juvenile *C. carpio* diets over 30 days. Growth performance, feed utilization, intestinal microvilli structure, gut microbiota, and amylase activity were analyzed using advanced techniques, including high performance liquid chromatography, scanning electron microscopy, and biochemical assays.

**Results:**

Kapok flower honey contains fructooligosaccharides (FOS, 14.76%) and inulin (6.6%). Supplementation at 1% significantly improved weight gain, feed conversion ratio, and specific growth rate. Amylase activity increased with honey supplementation, peaking at 24.13 ± 3.11 U g^−1^ protein for the 1% group. Gut morphology analysis revealed longer, denser intestinal microvilli and higher perimeter ratios in honey-treated groups than controls. Microbiota analysis showed increased beneficial *Bacillus* spp. exclusively in the honey-supplemented groups.

**Conclusion:**

Honey saccharides, particularly FOS and inulin, significantly enhance the growth performance, digestive enzyme activity, and gut health of common carp. Supplementation with 1% honey is optimal, improving feed efficiency and fostering beneficial gut microbiota. These findings highlight honey as a cost-effective, natural prebiotic for aquaculture.

## INTRODUCTION

The common carp (*Cyprinus carpio*) is one of the most widely cultivated fish globally, with an annual output exceeding 4.18 million metric tons and representing over 10% of total freshwater fish production worldwide [1]. As a consequence of its rapid growth, high egg production ability, and domestication by China over 8000 years ago, the common carp has recently become a leading choice for aquaculture cultivation [2]. The common carps are now bred for a variety of purposes, including food production, fishery restocking, ornamental use, and recreational fishing, in more than 100 countries where they have been introduced [3, 4]. However, commercial-scale carp farming can also encounter challenges, such as increased susceptibility to diseases due to increased stress levels and compromised immune systems. These diseases, which encompass bacterial diseases [5], viral [6, 7], and parasitic infections, can result in tremendous economic losses within the aquaculture sector [3]. One potential solution is the application of prebiotics, such as saccharides, in aquaculture systems to promote beneficial gut microbiota and improve immune function [8], thereby promoting disease resistance and growth performance [9].

Prebiotics play a significant role in supporting metabolic processes, maintaining health, and providing nutrients and energy to fish through anaerobic fermentation [10, 11]. The administration of saccharide prebiotics in aquatic animals affects the diversity of the gut microbiota, growth performance, immune response, and disease resistance [12–14]. Saccharides, including oligosaccharides (e.g., fructooligosaccharide [FOS], inulin, galactooligosaccharides [GOS], lactulose) and polysaccharides (e.g., chitin, pectin, starch, and hemicellulose), are resistant to hydrolysis by gastric acid. Instead, they are selectively fermented in the gut by beneficial bacteria [8, 9, 15]. As a result, FOS, inulin, oligofructose, and some of the dietary oligosaccharides facilitate an increase in the probiotic population and balance in the intestinal microbiota [16]. Meanwhile, polysaccharides also affect digestion and increase intestinal mass and the formation of short-chain fatty acids (SCFA), which affect intestinal health and digestion [17].

Honey contains glucose, fructose, and more than 30 oligosaccharides and has been identified as a potential natural prebiotic due to its richness in oligosaccharides and polysaccharides [18]. Dietary honey, a natural prebiotic, has been demonstrated to support the gastrointestinal microbiota and enhance fish health [19, 20]. Indonesia, a tropical country with a diverse range of honey-producing species [21], offers convenient and cost-effective access to honey for fish farming. Nevertheless, a paucity of research has examined the use of honey in common carp farming, particularly its effects on gut microbiota diversity and intestinal microvilli.

Given the dearth of research on the effects of honey on common carp farming, this study aimed to evaluate the effects of feeding common carp with honey on their growth performance, amylase enzyme activities, intestinal microvilli, and gut microbiota.

## MATERIALS AND METHODS

### Ethical approval

This study was conducted in accordance with the ethical guidelines for the treatment, care, and use of experimental animals as outlined in Minister of Marine Affairs and Fisheries Regulation Number 6/PERMEN-KP/2020 of 2020 concerning the Welfare of Cultured Fish. The ethical procedures of this study were conducted in compliance with the basic principles of experimentation under the supervision of Agricultural Faculty, Djuanda University (15/01/FAPERTA-B/IX/2023).

### Study period and location

The study was conducted from February to July 2023 at the Aquaculture Laboratory, Faculty of Agriculture, Djuanda University, Bogor, Indonesia.

### Analysis of the oligosaccharide content in honey

The study used Kapok (*Ceiba pentandra*) flower honey, which has been identified as the optimal type in previous studies [22]. Kapok flower honey was procured from Depok, West Java, Indonesia. The honey content was analyzed using high-performance liquid chromatography (HPLC). This analysis aimed to determine the type and concentration of polysaccharides present in the extracted prebiotics. The column utilized was the Sugar SP0810 (8.0 mm × 300 mm), with a particle size of 5 μL, equipped with a Waters® 2414 refractive index detector (Waters Corporation, USA), and operated at a flow rate of 2.0 mL/min. The mobile phase was a solution of acetonitrile and water for injection at a ratio of 80:20 acetonitrile: water. The sample volume injected was 20 μL with a column temperature of 40°C. The standard sugars used were FOS, inulin, kestose, and nystose. The formation of each peak indicates the presence of a specific sugar component. The retention time of each sugar component was then compared with that of the corresponding standard sugar. A similar retention time suggested that the component was of the same type as the standard.

### Rearing and dieting experiments to observe common carp growth performance

Four dietary treatments were employed: A control group (without honey supplementation) and honey-supplemented diets at doses of 0.5%, 0.75%, and 1% (g/kg of feed). These doses were determined based on the findings of previous studies conducted by Aryati *et al*. [22] and Fuandila *et al*. [23], while also considering the cost efficiency of the feed production process. Each treatment was conducted in quadruplicate. The commercial diet used in this study was a floating pellet (Hi-Pro Vite 781) with a protein content of 31%–33%, fat content of 4%–6%, and feed stability in water of 80% after 2 h. Diets containing honey were prepared by mixing the honey base with 2% hen egg albumen (as a binder) and then spraying the resulting mixture onto the diets. The diets were air-dried for 10–15 min before being administered to the fish.

A total of 320 juvenile common carp were used in this study, with an average total length of 11 ± 0.55 cm and an average body weight of 22.9 ± 0.72 g. The fish were obtained from the Djuanda University Aquaculture Laboratory in Bogor, West Java, Indonesia. The fish were allowed to acclimatize to their environment for seven days in a 500 L fiber tank before the diet was administered. The fish were then reared for 30 days in 16 units of 100 × 60 × 60 cm^3^ (60 L) aquariums with a stocking density of 20 fish per aquarium. They were fed three times a day: 7 a.m., 12 p.m., and 6 p.m. (GMT + 7). Synchronization and water exchange were performed each morning, and 10% of the total volume of water was removed from each aquarium. The water quality was maintained in accordance with the Indonesian National Standard, 2016, with a temperature range of 28°C–30°C, dissolved oxygen concentration of 4–6 mg/L, a pH level of 6.5–7.5, and total ammonia nitrogen (TAN) of 0.1–0.5 mg/L.

### Intestinal microvilli

On the 13^th^ day of the rearing period, three fish per tank (n = 12) were anesthetized using clove oil. The fish were carefully dissected, and the intestines were cut and rinsed with water (H_2_O). The intestinal specimens were then prepared in accordance with the methodology described by Goldstein *et al*. [24], which included the installation of the specimen on the stub, cleaning, prefixation, fixation, dehydration, drying, and Au coating using an ion coater. The cutting samples and subsequent observations were conducted in a vertical orientation. The observations were conducted using a JSM-IT200 InTouchScope™ scanning electron microscope (SEM) (JEOL Ltd, Japan). The ImageJ software (https://imagej.net/ij/download.html) was used to examine four images from each vertical cutting treatment, which were examined at a magnification of 4,000×. In accordance with the formula proposed by Dimitroglou *et al*. [25], the parameters measured were microvilli density (DMv) in arbitrary units, perimeter ratio (PR), and microvilli length (LMv) in micrometers (μm).

### Amylase enzyme activity

The amylase activity was quantified on day 30 of rearing. The intestines of five fish per tank (n = 20) were used for these analyses. The fish were anesthetized using clove oil and then dissected carefully. A 1 g sample of the intestine was then collected and homogenized in 10 mL of phosphate-buffered saline (8 g NaCl, 1.5 g Na_2_PO_4_, 0.2 g KCl, 0.2 g KH_2_PO_4_, and 1000 mL of distilled water). The intestines were homogenized and centrifuged at 27670× *g* for 20 min at 4°C. The resulting supernatant was used to analyze digestive amylase activity. Amylase activities were evaluated using the Bergmeyer–Grossi method [26].

### Intestinal microbiota composition

The intestinal microbiota composition was assessed on day 30 of rearing. Three fish per tank (n = 12) were anesthetized using clove oil and then carefully dissected. Intestinal content with a minimum weight of 5 g was collected and subjected to analysis, according to Cowan and Stell [27].

### Statistical analysis

All collected data were subjected to one-way analysis of variance (ANOVA) to evaluate the effects of different honey saccharide supplementation levels on the parameters measured. Following the ANOVA, Duncan’s multiple range test was applied for *post hoc* comparisons to identify significant differences between treatment groups. A significance level of p < 0.05 was used for all statistical analyses. The results are expressed as mean ± standard error. Statistical analyses were performed using SPSS version 16.0 for Windows (IBM Corp., NY, USA).

## RESULTS

### Oligosaccharide content in honey

Table 1 illustrates the oligosaccharide content of honey as a function of FOS and inulin. The FOS and inulin levels in Kapok flower honey were 14.76%, while the inulin levels were 6.6%. In addition, the kestose and nystose levels were below the limit of detection.

**Table 1:** Kapok honey content.

Oligosaccharide types	Kapok honey (%)
Fructo-oligosaccharides	14.76 ± 0.23
Inulin	6.60 ± 55
Kestose	< 0.37
Nystose	< 0.56

### Growth performance of common carp

The average survival rates across treatment groups were found to be 95%–98% and not significantly different (p > 0.05) among the treatment groups. The statistical analysis results of biomass weight (Wt) and weight gain (∆W) between treatments are presented in Table 2. The biomass weight and weight gain of common carp in the group with 1% honey saccharide exhibited the most notable and statistically significant difference (p < 0.05) compared to the other treatment groups. In contrast, the control group demonstrated the lowest value.

**Table 2:** Weight gain in common carp. Treatments with honey saccharide were as follows: 0.0% (control), 0.5%, 0.75%, and 1%.

Parameter	Control	Treatment		

		0.5%	0.75%	1.00%
W0 (g)	19.20 ± 1.25^a^	18.60 ± 0.16^a^	18.40 ± 0.09^a^	19.60 ± 0.05^a^
Wt (g)	31.28 ± 1.18^c^	36.0 ± 3.60^bc^	37.33 ± 2.50^b^	46.66 ± 3.08^a^
∆W (g)	12.08 ± 0.59^c^	17.4 ± 3.69^b^	18.93 ± 2.69^b^	27.06 ± 2.44^a^

Values in the same column with different superscript letters differ significantly (p<0.05). Data expressed as Mean ± standard error (n=6)

**Figure 1:**
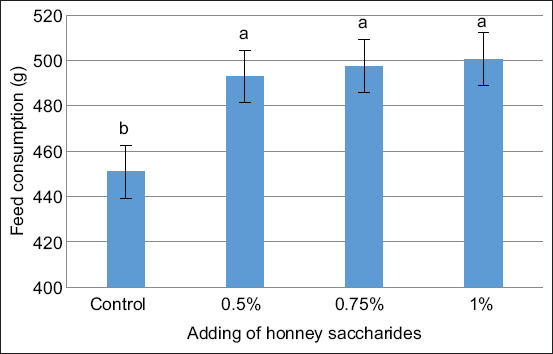
Feed consumption of common carp (*Cyprinus carpio*) juveniles fed with honey saccharides reared for 30 days. Treatments with honey saccharide were as follows: 0.0% (control), 0.5%, 0.75%, and 1%.

**Figure 2:**
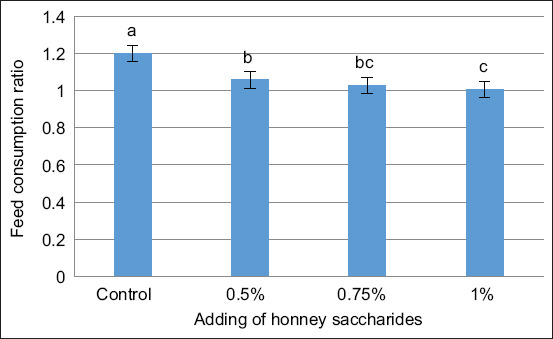
Feed conversion ratio of common carp (*Cyprinus carpio*) juveniles fed with honey saccharides reared for 30 days. Treatments with honey saccharide were as follows: 0.0% (control), 0.5%, 0.75%, and 1%.

**Figure 3:**
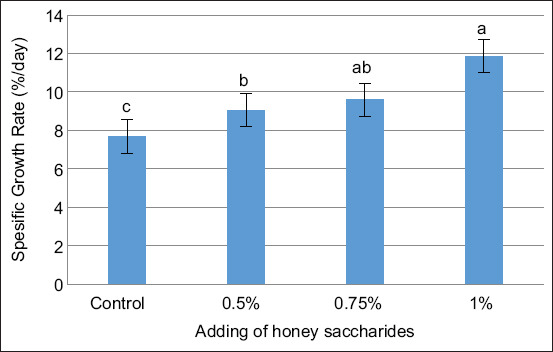
Specific growth rate of common carp (*Cyprinus carpio*) juveniles feeding on honey saccharides reared for 30 days. Treatments with honey saccharide were as follows: 0.0% (control), 0.5%, 0.75%, and 1%.

Figures 1–3 illustrate the impact of honey saccharides on feed consumption, feed conversion ratio (FCR), and specific growth rate of common carp, respectively. The feed consumption, feed conversion rate, specific growth rate, and all treatments exhibited a statistically significant difference (p < 0.05) compared with the control.

### Intestinal microvilli

**Figure 4:**
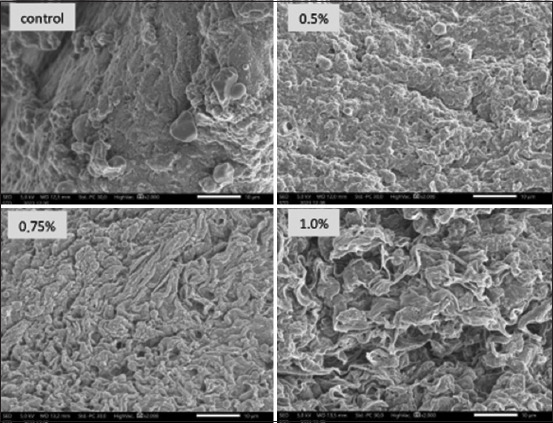
Gut microvilli of common carp (*Cyprinus carpio*) juveniles fed with honey saccharides reared for 30 days. Treatments with honey saccharide were as follows: 0.0% (control), 0.5%, 0.75%, and 1%.

Figure 4 illustrates the effect of administering honey saccharides through feeding on the intestinal microvilli of common carp. The observations revealed that the intestinal microvilli of common carp in the control group exhibited notable differences when compared to the treatment groups that received the addition of honey saccharides to the feed. The intestinal microvilli of common carp that were subjected to feed treatment with honey incorporation were longer and denser than those observed in the control group.

LMv, PR, and DMv in common carp supplemented with honey saccharides using SEM are presented in Table 3. The intestinal microvilli of common carp in the control group exhibited notable differences compared to those in the treatment groups supplemented with honey saccharides. The LMv, perimeter, and density in all treatment groups were significantly different (p < 0.05) compared with the control group.

**Table 3:** LMv, PR, and DMv of the intestines of common carp fed honey saccharides. Treatments with honey saccharide were as follows: 0.0% (control), 0.5%, 0.75%, and 1%.

Parameter	Control	Addition of honey saccharides		

		0.5%	0.75%	1.00%
LMv (mm)	8.00 ± 0.83^b^	8.11 ± 0.78^a^	8.2 ± 0.22^a^	8.55 ± 0.34^a^
PR (AU)	2.23 ± 0.16^b^	3.52 ± 0.15^a^	3.54 ± 0.38^a^	3.51 ± 0.11^a^
DMv (AU)	3.74 ± 0.19^c^	6.24 ± 0.13^b^	6.49 ± 0.01^a^	6.70 ± 0.10^a^

Values in the same column with different superscript letters differ significantly (p<0.05). Data expressed as Mean ± standard error (n=6) LMv=Microvilli length, PR=Perimeter ratio, DMv=Microvilli density, AU=Arbitrary units

### Amylase enzyme activity

**Figure 5:**
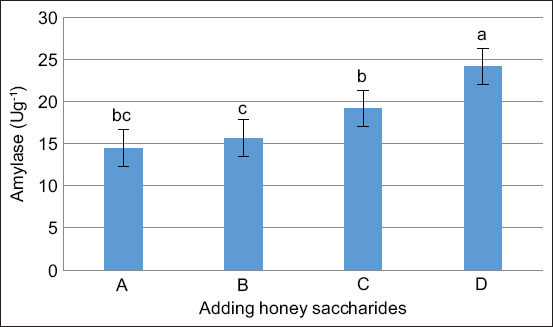
Amylase enzyme in the gastrointestinal tract of common carp (*Cyprinus carpio*) juveniles fed with honey saccharides reared for 30 days. Treatments with honey saccharide were as follows: 0.0% (control), 0.5%, 0.75%, and 1%.

Figure 5 illustrates the activity of amylase in common carp after 30 days of honey saccharide administration. The amylase enzyme activity in the treatment group with 1% honey dose exhibited the highest value. It was significantly different (p < 0.05) compared with the groups with lower doses of treatment and the control group.

### Intestinal microbiota composition

**Figure 6:**
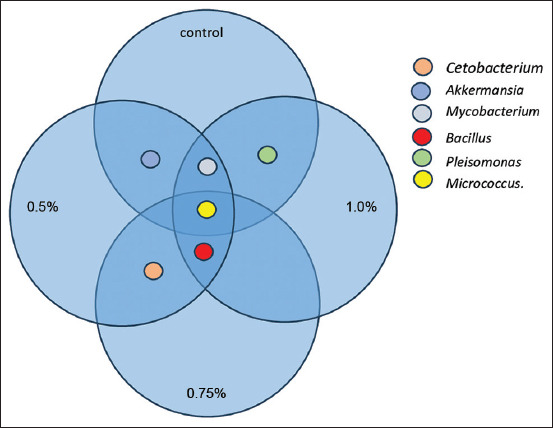
Venn diagram of the microbiota of common carp (*Cyprinus carpio*) juveniles feeding on honey saccharides reared for 30 days. Treatments with honey saccharide were as follows: 0.0% (control), 0.5%, 0.75%, and 1%.

The Venn diagram of the administration of honey saccharides to common carp is shown in Figure 6. The control group found the following genera: *Cetobacteriu*m, *Akkermansia*, *Mycobacterium*, *Micrococcus*, and *Pleisomonas*. The treatment group administered 0.5% honey saccharides exhibited the presence of the genera: *Cetobacterium*, *Akkermansia*, *Mycobacterium*, *Bacillus*, and *Micrococcus*. The treatment group receiving 0.75% honey saccharides exhibited the presence of *Mycobacterium*, *Bacillus*, and *Micrococcus*. The group treated with 1% honey saccharides exhibited the genera *Cetobacterium*, *Bacillus*, *Micrococcus*, and *Plesiomonas*.

## DISCUSSION

The present study employed honey, which has been demonstrated to be beneficial in aquaculture, for example, as an enhancer of survival and growth performance or as an immunostimulant [12, 13, 20, 22]. The administration of honey to Nile tilapia (*Oreochromis niloticus*) has shown that dietary supplementation of up to 1% honey can improve growth performance, reduce the FCR, increase digestive enzyme activities, and optimize the intestinal microbiota [22]. Furthermore, a study conducted by Fuandila *et al*. [23] on *Penaeus vannamei* demonstrated a comparable outcome wherein the administration of 0.75% honey in the diet enhanced growth performance, immune response, and resistance to the pathogen *Vibrio parahaemolyticus*.

Honey contains various saccharides, including monosaccharides, disaccharides, oligosaccharides, and polysaccharides [28]. It is considered a multi-prebiotic material, and its active ingredients include FOS [29], xylo-oligosaccharide [30], GOSs, and inulin [16]. In addition, honey contains minerals, vitamins, free amino acids, organic acids, flavonoids, phenolic compounds, and aromatic substances. The chemical composition of honey depends on the plant from which it was collected and its geographical origin [31].

The Kapok flower honey used in the present study contained prebiotics, namely FOS (14.76%) and inulin (6.60%) (Table 1). FOSs are a class of oligosaccharides whose monomers are often referred to as neosugars. These consist of short chains of D-glucose and D-fructose, typically with 3–5 monosaccharide units [32, 33]. FOSs are a non-digestible carbohydrate that provides prebiotic properties that stimulate the growth of beneficial gut flora, improve digestion processes, and enhance immune function [34]. Inulin, another type of oligosaccharide, is a fructose polymer that serves as a nutrient for beneficial bacteria in the gut [35]. Gen*ç et al*. [36] and Paz *et al*. [37] reported that FOS supplementation at concentrations of 0.1% and 0.5% have beneficial effects on the growth and health of the tambaqui *(Colossoma macropomum*). In addition, FOS improved the length, weight, and survival rates of *P. vannamei* post-larvae and boosted immune responses through increased phenoloxidase, lysozyme, and lectin activities [38]. Furthermore, Yones *et al*. [39] observed that inulin supplementation at a dose of 2.5 g/kg improved growth performance, immune indices, parasitic resistance, liver, and spleen structure in Nile tilapia (*O. niloticus*) fingerlings. In addition, Genç *et al*. [36] found that adding mannanoligosaccharides to the diet of African catfish (*Clarias gariepinus*) positively affected growth parameters and improved health conditions.

In addition to using honey as a prebiotic, numerous studies [40–44] have also focused on pursuing novel food supplements that improve fish growth and immunity. A substantial number of studies have yielded comparable results, indicating that the incorporation of natural ingredients into the diet of fish and other animals exerts a beneficial influence on their overall physiological well-being. The administration of β-1,3/1,6-glucan to common carp (*C. carpio*) resulted in the formation of a more diverse intestinal community, which may serve as a barrier against the invasion and establishment of pathogenic microorganisms [40]. In addition, β-glucan is an effective agent for protecting against the genotoxicity induced by aflatoxin B1 and effectively alleviating the lesions caused by aflatoxin B1. The supplementation of betaine (0.3%) has been shown to significantly increase the growth and survival rates of common carp (*C. carpio*) [41]. The administration of GOS to common carp has been demonstrated to positively affect fish physiology and the development of the gastrointestinal tract [42]. A study by Arciuch-Rutkowska *et al*. [43] employed next-generation sequencing metagenomic analysis to evaluate the effects of dietary supplementation with sodium butyrate, β-glucan, and vitamins on the gut microbiome diversity of sturgeon hybrids (*Acipenser gueldenstaedtii*♀ × *Acipenser baerii*♂). The results revealed that the supplemented group exhibited the highest species richness and α-diversity index compared to the control group. Notably, probiotic bacteria such as *Lactobacillus* and *Lactococcus* were present at higher proportions in the supplemented groups. Similarly, Arciuch-Rutkowska *et al*. [44] reported that dietary supplementation with sodium butyrate, β-glucan, and vitamin preparation (FishQuatro) had an impact on the growth performance and gut microbiota of juvenile African catfish (*C. gariepinus*). These supplements enhanced growth parameters and positively influenced the composition of the gastrointestinal microbiota. An increase in beneficial probiotic bacteria, including *Lactococcus* and *Lactobacillus*, was observed. In contrast, potentially harmful bacteria, such as *Candidatus Arthromitus*, were significantly reduced in the supplemented groups compared with the control group.

In the large intestine, prebiotics are fermented by the gut microbiota to produce SCFAs, which in turn supply energy to intestinal cells and help maintain a strong intestinal barrier as well as exhibit anti-inflammatory effects [15]. The FCR was significantly lower (p < 0.05) in all groups of honey prebiotic treatment compared with the control group. This indicates better utilization of feed in the gut, a more balanced microbiota, elevated enzyme activity due to probiotic bacteria, and higher intestinal functionality [45]. The addition of honey to the feed enhances the diversity and evenness of the gastrointestinal microbiota involved in nutrient digestion. By fermenting the oligosaccharides, probiotic bacteria provide a growth substrate that elicits inhibitory effects against pathogenic colon bacteria [19] as well as produce active compounds (i.e., enzymes, amino acids, vitamins) that ultimately contribute to increased nutrient uptake, feed utilization, and digestion in the host [45].

Microvilli are microscopic, finger-like extensions of the surface of epithelial cells, for example, in the small intestine [46, 47]. They contribute to an increase in the surface cell layer, which enhances nutrient absorption, digestion efficiency, and substance transport. In common carp, males exhibited a longer body length than females. Feeding with honey saccharides resulted in a significant increase in the length of microvilli in the intestine, with the highest observed length occurring in fish fed high doses of honey saccharides. The PR was greater in the honey-fed group than in the control group, suggesting an increase in villi development. This increases the enterocytic absorptive surface and improves nutrient absorption [48]. Improved gut morphology is an important factor in increasing growth performance and represents a sign of cost-effective feed utilization. This finding is consistent with previous studies on other fish species, such as tilapia, *O. niloticus* (Linnaeus, 1758) [49], and red drum (*Sciaenops ocellatus*) [50].

Regarding enzyme activity, this experiment demonstrated that amylase activity was elevated in the presence of higher concentrations of honey saccharides (Figure 5). The observed increase in amylase activity indicates improved gastrointestinal function. The gut microbiota produces exogenous enzymes to compensate for the absence of endogenous enzymes and neutralize the anti-nutritional factors in the diet, thereby promoting increased growth performance [51]. One such enzyme is amylase, which plays an essential role in the digestive process within the intestine by hydrolyzing starch and aiding digestion [45]. As a result, high levels of amylase activity were observed in the groups treated with 1% honey saccharide (24.13 ± 3.11 U/g protein), which also exhibited the greatest improvement in amylase activity. This is likely due to the higher concentration of oligosaccharides present in honey, which provide a substrate for the stimulation of exogenous enzyme production by gut microflora.

The role of anaerobic fermentation by gut microbiota in carbohydrate fermentation, which converts indigestible feed into SCFAs, is of significant importance [45, 52]. In aquaculture, SCFAs have been demonstrated to significantly affect growth performance [53] and immune function [15]. Analysis of bacterial samples revealed the presence of *Bacillus* genus exclusively within the honey treatment groups, with no detection in the control group. Some *Bacillus* species are recognized as probiotics in aquaculture due to their ability to enhance feed digestibility, prevent microbial diseases, reduce water pollution, and survive harsh environmental conditions while remaining nonpathogenic and nontoxic to fish [54, 55]. Honey contains prebiotics and sugars, which have been demonstrated to increase probiotic bacterial populations, alter the microbiota composition, and improve the growth performance and gut morphology of tilapia [56]. The prebiotics demonstrated broad-spectrum antibacterial activity against Gram-positive and Gram-negative bacteria, including antibiotic-resistant strains such as methicillin-resistant *Staphylococcus aureus*. In addition, they improved the adhesion of beneficial bacteria, such as bifidobacteria, within the fish gut, thereby inhibiting the colonization of pathogenic bacteria [57, 58].

**Figure 7:**
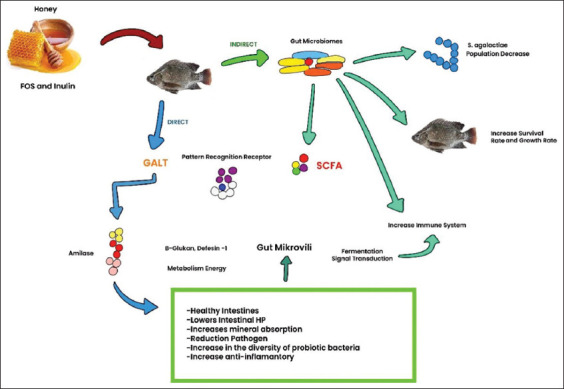
The purpose of feeding fish honey saccharides.

A schematic model illustrating the growth performance mechanism of common carp-fed honey saccharides is presented in Figure 7. Honey saccharides function as prebiotics through two distinct pathways: Direct and indirect. Prebiotic molecules directly interact with intestinal microvilli in the direct pathway, resulting in increased digestive enzyme activity. In the indirect pathway, prebiotic molecules interact with the gut microbiota, subsequently affecting fish. Further research is required to evaluate the effect of honey as a prebiotic on the immune system of fish and the effectiveness and efficiency of honey as a prebiotic in large-scale field and multilocation tests.

## CONCLUSION

This study demonstrates that Kapok flower honey saccharides, rich in FOS and inulin, serve as an effective natural prebiotic for enhancing the growth performance, digestive enzyme activity, intestinal morphology, and gut microbiota diversity in common carp (*C. carpio*). Among the supplementation levels, 1% honey exhibited the most pronounced effects, significantly improving specific growth rate, FCR, and amylase activity, as well as promoting the development of longer and denser intestinal microvilli. In addition, the presence of beneficial Bacillus species in the gut microbiota of honey-treated groups underscores its potential to enhance gut health and overall fish vitality.

This study provides novel insights into the use of honey saccharides as a cost-effective and sustainable prebiotic in aquaculture. The robust experimental design, employing advanced analytical techniques such as HPLC, SEM, and microbiota analysis, ensures the reliability and reproducibility of the findings. The comprehensive evaluation of growth, digestive, and microbiological parameters highlights the multifaceted benefits of honey supplementation in aquaculture systems.

While the study effectively demonstrates the benefits of honey saccharides, it does not evaluate their impact on the immune system or disease resistance of common carp, which is critical aspects of aquaculture sustainability. In addition, the study was conducted in a controlled laboratory environment, limiting its applicability to field-scale aquaculture systems.

Future studies should explore the long-term effects of honey supplementation on fish health and immune response under field conditions. The potential synergistic effects of honey saccharides with other prebiotics or probiotics should also be investigated. Furthermore, large-scale trials in commercial aquaculture setups are necessary to confirm the cost-effectiveness and scalability of using honey saccharides as a dietary additive. This study provides a foundation for the broader application of natural prebiotics in sustainable aquaculture practices.

## AUTHORS’ CONTRIBUTIONS

YA: Conceptualization, data curation, formal analysis, investigation, and methodology. EF: Data curation, formal analysis, project administration, visualization, and drafted, reviewed, and edited of the manuscript. LS: Conceptualization, methodology, and investigation. LG: Data curation, formal analysis, methodology, and visualization. IK: Data curation, resources, formal analysis, and drafted, reviewed, and edited the manuscript. ES: Validation of data, supervision, and edited the manuscript. MY: Supervision, validation of data, and drafted the manuscript. PO: Administration of the research project, methodology, and edited the manuscript. DP: Supervision and edited the manuscript. DS: Validation of data and reviewed and edited the manuscript. All authors have read and approved the final manuscript.
